# Supplementary material for: Sex as Biological Variable in Cardiac Mitochondrial Bioenergetic Responses to Acute Stress
Source: Int J Mol Sci. 2022 Aug 18;23(16):9312. doi: 10.3390/ijms23169312 (PMC9409303; doi:10.3390/ijms23169312)
Supplement: Supplementary file 1 [file ijms-23-09312-s001.zip › ijms-1829397-supplementary.pdf]

**Supplemental Figure S1.**

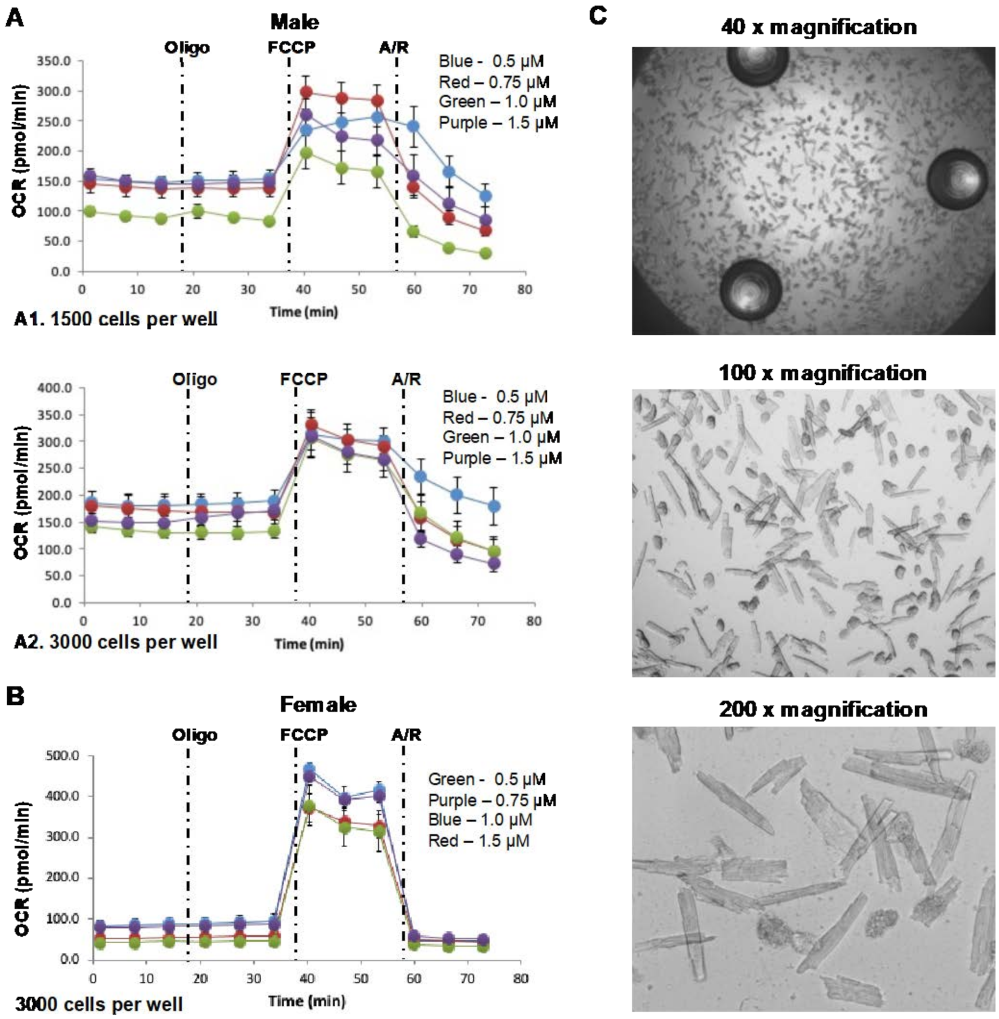


**Figure S1.** Metabolic profiling trace following addition of different FCCP concentration in cardiomyocytes. **A, B.** The OCR trace of different groups with 0.5, 0.75, 1, and 1.5 μM of FCCP at cell density of 1500 **(A1)** and 3000 cells per well **(A2, B)** in cardiomyocytes isolated from male (**A**) and female (**B**) adult mice. **C.** Representative images of cardiomyocytes in laminin-coated Seahorse V3 96-well culture plate prior to assay in different magnifications.

**Supplemental Figure S2.**

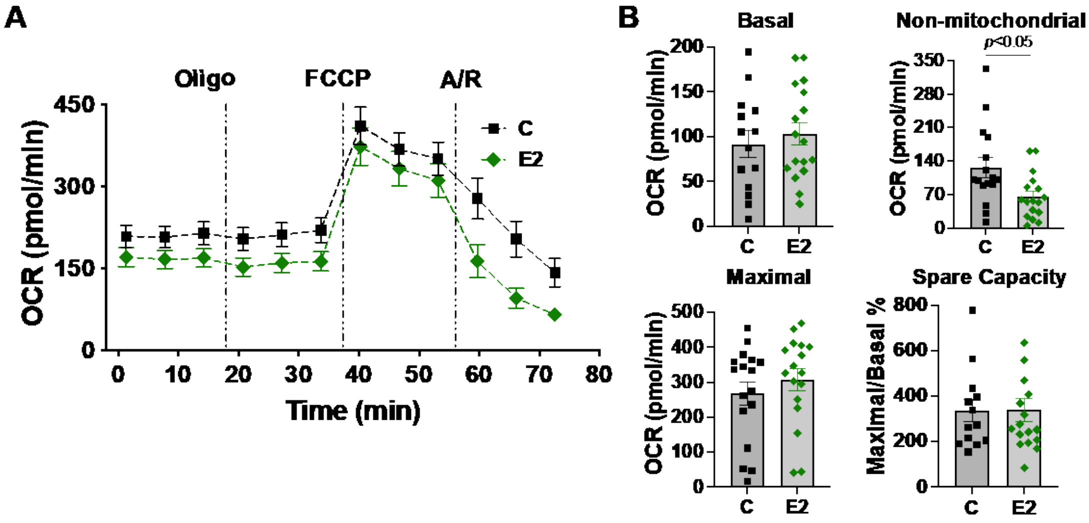


**Figure S2.** Effect of E2 on bioenergetics in cardiomyocytes from male adult mice without stress. **A.** The OCR trace of adult cardiomyocytes in the absence or presence of E2 (100 nM). **B.** Quantification of basal OCR, non-mitochondrial OCR, maximal rate, and spare capacity in E2-treated cardiomyocytes from male adult mice.

**Supplemental Figure S3.**

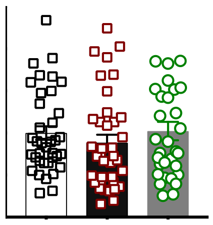

**Figure S3.** Effects of E2 on mitochondrial membrane potential in female cardiomyocytes upon TNFα exposure. Mitochondrial membrane potential was quantified by the ratio of red vs. green fluorescence intensity in JC-1-stained cardiomyocytes from female adult mice.

**Supplemental Figure S4.**

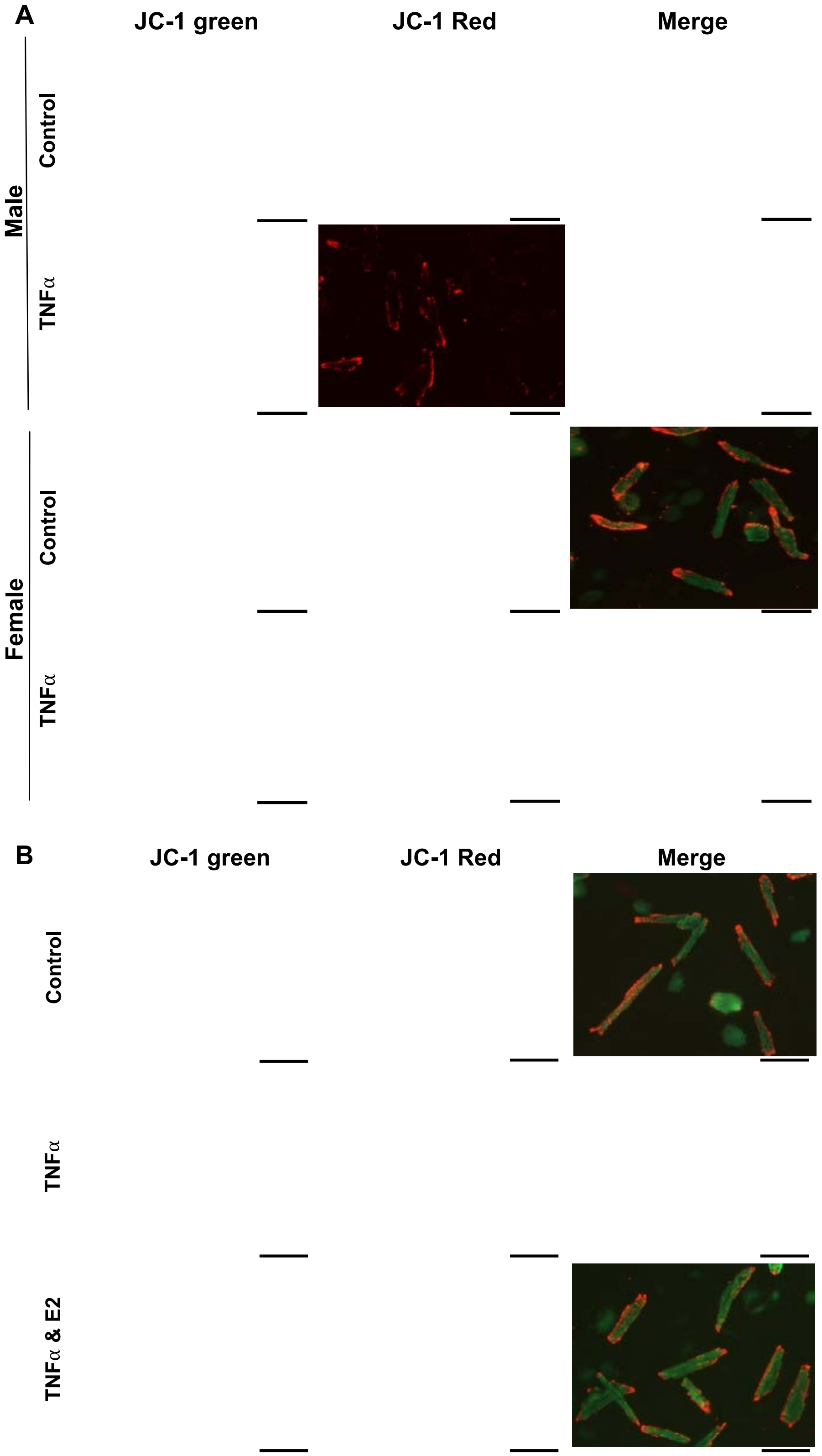

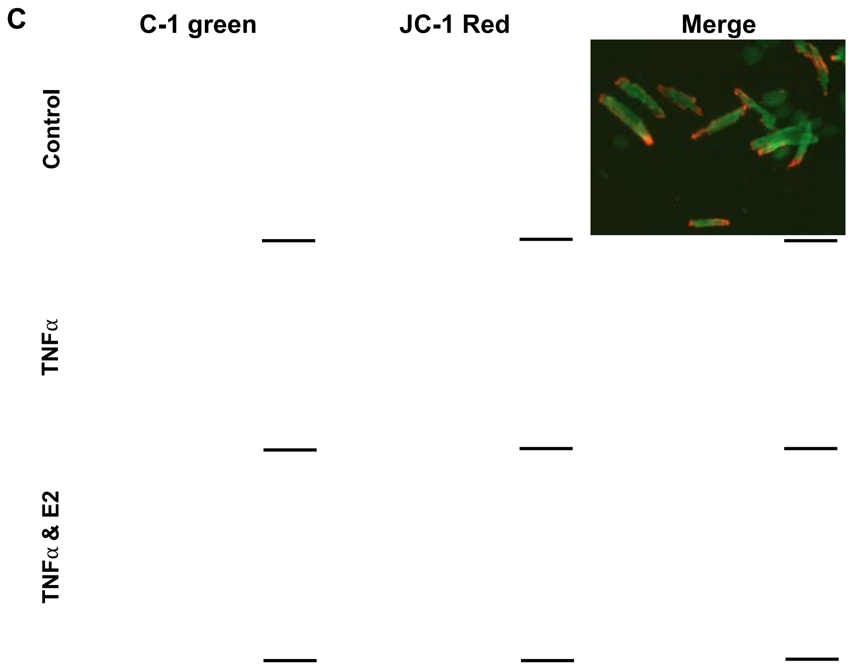


**Figure S4.** Representative images of JC-1-stained cardiomyocytes in each experimental group. **A.** JC-1-stained cardiomyocytes from male and female adult mice upon TNFα exposure. **B.** JC-1-stained male cardiomyocytes without or with TNFα and E2 treatment. **C.** JC-1-stained female cardiomyocytes +/- TNFα and E2 treatment. Scale bar = 100 μm. Fluorescence intensity of red and green was quantified in separated individual cardiomyocyte using ImageJ.

**Supplemental Figure S5.**

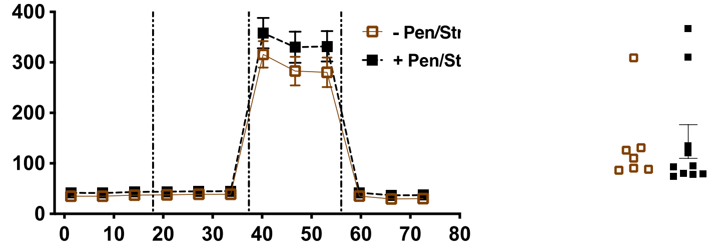


**Figure S5.** Effect of Pen/Strep on mitochondrial respiration in adult cardiomyocytes. **A.** The OCR trace of adult cardiomyocytes in the absence or presence of 1% Pen/Strep in culture media. **B.** Quantification of basal OCR, non-mitochondrial, and maximal OCR in cardiomyocytes +/- 1% Pen/Strep. Cardiomyocytes were isolated from male and female adult mice and plated in laminin-precoated Seahorse V3 96-well microplate at 3000 cells/well in culture media +/- 1% Pen/Strep. After 2 hours, the cells were used for Seahorse XF Cell Mito Stress test. Similar results were observed between cardiomyocytes from male and female mice. Shown are the mitochondrial metabolic results combined from male and female cardiomyocytes.
